# Enhanced alloresponse to platelet transfusion due to immune dysregulation following ablative chemotherapy in mice

**DOI:** 10.3389/fimmu.2023.1281123

**Published:** 2023-11-27

**Authors:** Rachael P. Jackman, Orsolya Darst, Betty Gaillard, Johnson Q. Tran, Mary M. Tomayko, Marcus O. Muench

**Affiliations:** ^1^ Vitalant Research Institute, San Francisco, CA, United States; ^2^ Department of Laboratory Medicine, University of California, San Francisco, San Francisco, CA, United States; ^3^ Department of Dermatology, Yale School of Medicine, New Haven, CT, United States; ^4^ Department of Pathology, Yale School of Medicine, New Haven, CT, United States

**Keywords:** platelets, alloimmunization, chemotherapy, recipient health, alloantibodies, transitional B cells, T cells

## Abstract

**Introduction:**

Alloimmunization is common following platelet transfusion and can result in negative outcomes for recipients such as refractoriness to subsequent transfusions and rejection of transplants. Healthy people do not receive blood transfusions, and the diseases and therapies that result in a need to transfuse have significant impacts on the immunological environment to which these alloantigens are introduced. Ablative chemotherapies are common among platelet recipients and have potent immunological effects. In this study, we modeled the impact of chemotherapy on the alloresponse to platelet transfusion. As chemotherapies are generally regarded as immunosuppressive, we hypothesized that that they would result in a diminished alloresponse.

**Methods:**

Mice were given a combination chemotherapeutic treatment of cytarabine and doxorubicin followed by transfusion of allogeneic platelets, and compared to controls given no treatment, chemotherapy alone, or transfusion alone. Alloantibody responses were measured 2 weeks after transfusion, and cellular responses and growth factors were monitored over time.

**Results:**

Contrary to our hypothesis, we found that chemotherapy led to increased alloantibody responses to allogeneic platelet transfusion. This enhanced response was antigen-specific and was associated with increased CD4^+^ and CD8^+^ T cell responses. Chemotherapy led to rapid lymphocyte depletion followed by reconstitution, non-specific activation of transitional B cells with the highest levels of activation in the least mature subsets, and increased serum levels of B cell activating factor (BAFF).

**Conclusion:**

These data suggest that ablative chemotherapy can increase the risk of alloimmunization and, if confirmed clinically, that additional measures to protect these patient populations may be warranted.

## 1 Introduction

Allogeneic transfusion, while commonplace, represents a major immunological event, potentially exposing the recipient to a range of foreign antigens. Many platelet recipients become alloimmunized, commonly against donor major histocompatibility complex (MHC) antigens, with significant potential consequences such as platelet refractoriness and transplant rejection (1–6). Measured frequencies of anti-MHC alloimmunization vary widely between studies with estimates ranging from 7-55% (1, 7–14).

Several factors may contribute to this wide range in measured frequencies of alloimmunization. While much progress has been made on reducing the immunogenicity of platelet products through modern blood-banking practices such as leukoreduction and using crossmatched or human leukocyte antigen (HLA) matched platelets in refractory recipients, little focus has been given to the immunological environment of the recipient. Healthy individuals are not transfused, and the diseases and treatments that necessitate transfusion also impact host immunity, often in significant ways. Approximately 44% of hospital platelet usage within the United States is within Hematology/Oncology/Bone Marrow Transplant services (15), and many of these recipients receive ablative chemotherapies. These therapies can deplete both platelets and lymphocytes, generating both a need for allogeneic transfusion and an altered immune setting to which these alloantigens are introduced (16–20). Evaluating the contribution of these therapies to platelet alloimmunization clinically is challenging as many factors that contribute to the alloresponse such as platelet dosing, types of platelet products used, and underlying disease, are interconnected. Animal models offer the opportunity to dissect these variables under defined and controlled conditions.

Ablative therapies such as chemotherapy and radiation are generally thought of as immunosuppressive, wiping out portions of the immune system, and rendering recipients more vulnerable to infection (21). Following ablation, homeostatic regulation of the immune system during recovery can further alter immunity, creating circumstances for robust, albeit dysregulated, immune responses. Reductions in cytokine consumption can result in increased levels of circulating homeostatic growth factors available to remaining lymphocytes (22, 23). Early T cell reconstitution is dominated by remaining mature T cells rather than thymopoiesis (22, 24), which in the presence of foreign antigen can result in over-expansion of antigen-specific T cells (25, 26). Initial B cell reconstitution is dominated by transitional B cell subsets (T1, T2, and T3) (27–29). These cells have not been subjected to the same degree of negative selection as their more mature counterparts, and they respond differently to B cell receptor (BCR) signaling than mature B cells (28–31). These competing forces of ablation and permissive expansion could have a profound impact on the immune response to allogeneic transfusion.

In this study, we utilized a murine model to assess the contribution of ablative chemotherapy on the immune response to allogeneic platelet transfusion, hypothesizing that this immunological environment would suppress the alloantibody response. Utilizing an acute myeloid leukemia (AML) chemotherapy model developed by Zuber et al. (32), combined with our own model of platelet alloimmunization (33–37), we have determined the impacts of ablative chemotherapy on alloimmunization outcomes and the immune environment at the time of transfusion.

## 2 Materials and methods

### 2.1 Mice

C57Bl/6J female mice, between 2-3 months old were used as recipients, and a mixture of male and female BALB/cJ mice between 2-6 months were used as donors. Mice were purchased from The Jackson Laboratory and maintained under barrier conditions in a specific-pathogen-free vivarium at Vitalant Research Institute. All studies were conducted with approval and oversight by the Institutional Animal Care and Use Committee at Labcorp Early Development Laboratories Inc. (San Carlos, CA) under Animal Welfare Assurance A3367-01. The recommendations in the Guide for the Care and Use of Laboratory Animals of the National Institutes of Health were followed for animal husbandry. Mice were housed in sterile, single-use microisolator cages containing either corn-cob or Alpha-Dri bedding (Innovive Inc.). Mice were given free access to sterile-filtered, acidified water (Innovive Inc.), and sterile, irradiated Teklad Global 19% protein diet (Envigo). Cages were enriched with autoclaved cotton Neslets (Ancare Corp.) and GLP-certified Bio-Huts (Bio-Serv).

### 2.2 Chemotherapy and transfusion

Chemotherapy was administered via intraperitoneal injection and included 100mg/kg cytarabine (Covetrus) + 3mg/kg doxorubicin (Fisher BioReagents) daily for 3 days (days 0-2) followed by 2 additional days of 100mg/kg cytarabine alone (days 3-4) as described by Zuber et al. (32) Non-leukoreduced platelet rich plasma (PRP) was prepared from fresh BALB/cJ donors as previously described (34), producing PRP with mean platelet counts of 4.40x10^8^/mL (4.40x10^7^/transfusion) and mean white blood cell counts of 5.24x10^6^/mL (5.24x10^5^/transfusion). 100μL of prepared PRP was administered intravenously on day 7.

### 2.3 Sample collection

Non-terminal whole blood samples for complete blood counts (CBC) were collected via tail clip into 20μL Minivette POCT K3 EDTA tubes (Sarstedt). Terminal blood samples for measurement of antibodies and cytokines were collected via orbital enucleation, allowed to clot for 20-30 minutes, then separated by centrifugation, with serum aliquoted and stored at -80°C. Spleens were processed by mincing, straining through 100-μm cell strainer (Becton Dickinson, BD Falcon), lysis of red blood cells (RBCs), washing, and counting.

### 2.4 Antibody screening

Anti-donor antibodies were measured in the serum of recipient mice by flow cytometry using BALB/cJ splenic T cells as target cells. Splenocytes were first incubated with anti-FcR blocking antibody (2.4G2), washed, then incubated with serum diluted 1:16 in phosphate buffered saline. Cells were then washed and stained with antibodies against TCRβ (PerCP-Cy5.5, H57-597), B220 (AlexaFluor700, RA3-6B2), Igκ (APC-Cy7, RMK-45), IgM (PE-Cy7, RMM-1), IgG1 (AlexaFluor488, RMG1-1), IgG2b (PE, RMG2b-1), IgG2a (used here to detect IgG2c as it is cross-reactive) (APC, RMG2a-62) (Biolegend); IgG3 (BV421, R40-82) (BD Biosciences), washed, then run on a spectral flow cytometer (Cytek Aurora), and analyzed with FlowJo (FlowJo, LLC). Cells were gated on lymphocytes, singlets, then B220^-^/TCRβ^+^ T cell events. Median fluorescent intensity for each isotype was measured within this population.

Total serum antibody levels were measured using the MILLIPLEX Mouse Immunoglobulin Isotyping Magnetic Bead Panel (Millipore Sigma), running on the Luminex 200 platform (Luminex), and analyzing with Bio-Plex Manager (Bio-Rad). Samples were run in duplicate on lot matched kits according to manufacturer’s instructions.

### 2.5 Cytokine screening

Serum was screened for B cell activating factor (BAFF) by enzyme-linked immunosorbent assay (ELISA) using the Mouse BAFF/BLyS/TNFSF13B Immunoassay (R&D Systems) according to manufacturer’s instructions using a 1:20 serum dilution. IL-7 and IL-15 were measured using the MILLIPLEX MAP Mouse Cytokine/Chemokine Magnetic Bead Panel (Millipore Sigma), and IL-21 with the MILLIPLEX MAP Mouse TH17 Magnetic Bead Panel (Millipore Sigma) according to manufacturer’s instructions.

### 2.6 Cell screening

**Figure 1:**
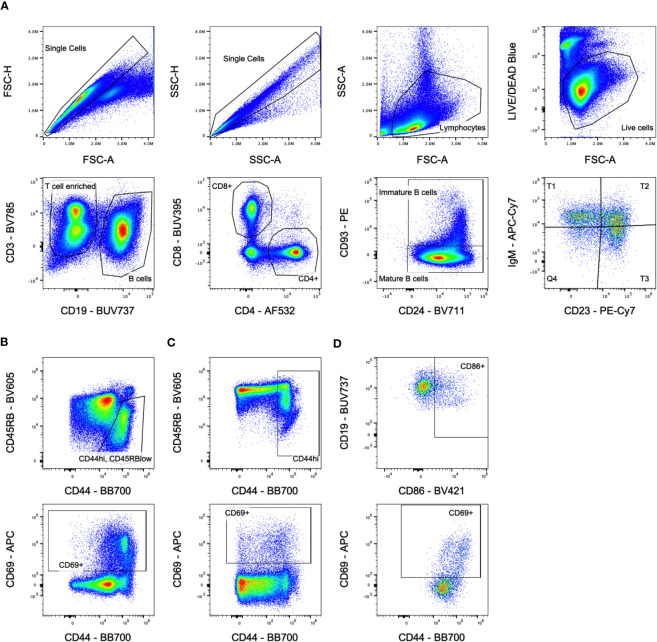
Flow cytometry gating. **(A)** Overall population gating is shown. Cells were gated on singlets, lymphocytes, live cells, then either CD3^+^/CD19^-^ for T cells or CD3^-^/CD19^+^ for B cells. T cells were further gated on CD4^+^ or CD8^+^ populations, and B cells were gated on mature CD93^-^/CD24^lo^ or immature/transitional CD93^+^/CD24^hi^ populations. Immature B cells were subdivided into T1, T2, and T3 populations based on CD23/IgM expression. Sample shown is from the untreated group at day 5. **(B)** Activation marker gates for CD4^+^ T cells. Sample shown is from the transfusion only group at day 14 for the CD44^hi^/CD45RB^low^ gate and day 10 for the CD69^+^ gate. **(C)** Activation marker gates for CD8^+^ T cells. Sample shown is from the transfusion only group at day 14 for the CD44^hi^ gate and day 10 for the CD69^+^ gate. **(D)** Activation marker gates for B cells. Sample shown is from the chemo only group at day 5 gating on immature B cells.

CBCs were run on the HT-5 (Heska) according to manufacturer’s instructions. For analysis of splenocytes, cells were first incubated with anti-FcR blocking antibody (2.4G2), washed, stained with LIVE/DEAD™ Fixable Blue Dead Cell Stain Kit (ThermoFisher Scientific), washed, then stained with antibodies against CD3 (BV785, 145-2C11), CD69 (APC, H1.2F3), CD93 (PE, AA4.1), CD86 (BV421, GL-1), CD23 (PE-Cy7, B3B4), IgD (AlexaFluor700, 11-26c.2a), IgM (APC-Cy7, RMM-1), (Biolegend); CD4 (AlexaFluor532, RM4-5), (eBioscience); CD8a (BUV395, 53-6.7), CD44 (BB700, IM7), CD45RB (BV605, 16A), H-2D^d^ (FITC, 34-3-12), H-2D^b^ (BV480, KH95), CD19 (BUV737, 1D3), IA/IE (BUV805, 2G9), and CD24 (BV711, M1/69), (BD Biosciences). Cells were washed and fixed, then run on a spectral flow cytometer (Cytek Aurora), and analyzed with FlowJo (FlowJo, LLC). Gating is shown in **Figure 1**. Cells were gated on singlets, lymphocytes, live cells, then either CD19^-^ to enrich for T cells or CD3^-^/CD19^+^ for B cells. T cells were further gated on CD4^+^ or CD8^+^ populations, and B cells were gated on mature CD93^-^ or immature/transitional CD93^+^ populations. Immature B cells were subdivided into T1 (CD23^-^/IgM^hi^), T2 (CD23^+^/IgM^hi^), and T3 (CD23^+^/IgM^low^) populations.

### 2.7 Statistical analysis

Graphing and analysis were completed using Prism version 9 (GraphPad Software). Unpaired t-tests were used for comparisons between 2 groups. For comparisons between more than 2 groups, one-way Analysis of Variance (ANOVA) with Tukey’s post-test for multiple comparisons was used to compare each group to all other groups.

## 3 Results

**Figure 2:**
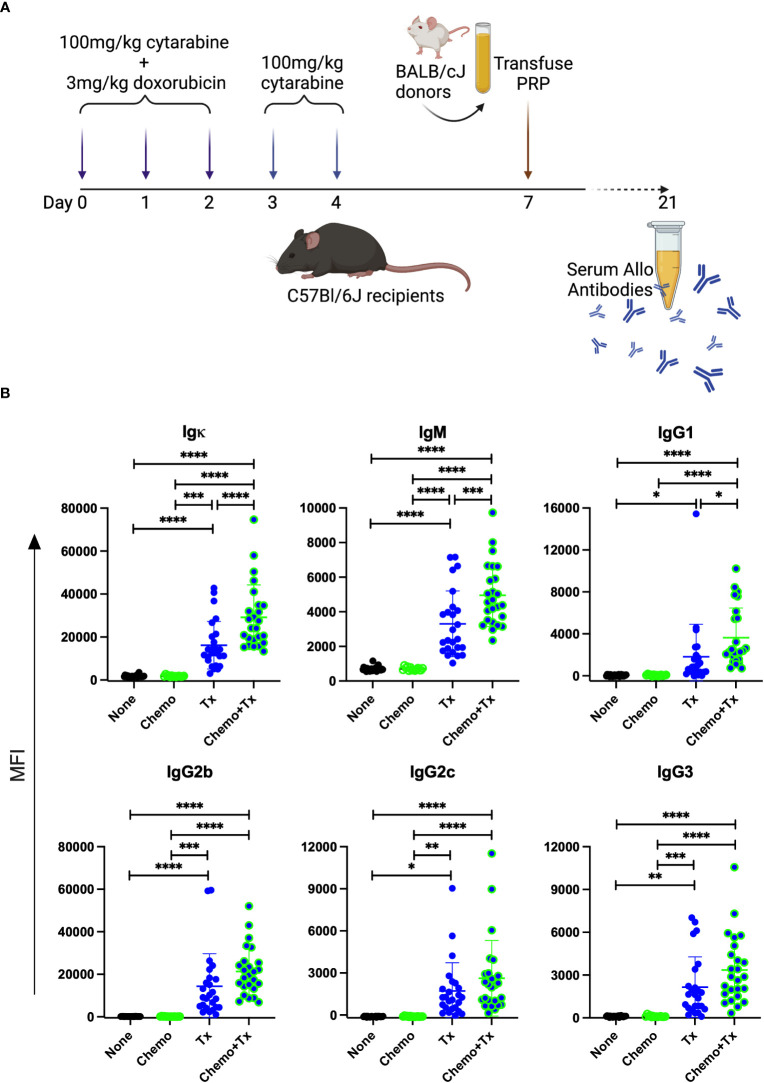
Chemotherapy leads to enhanced alloantibody response to platelet transfusion. **(A)** C57Bl/6J mice were given either no treatment (n=25) (None), a 5-day course of cytarabine/doxorubicin days 0-4 (n=14) (Chemo), a BALB/cJ platelet transfusion at day 7 (n=25) (Tx), or the chemotherapy on days 0-4 followed by transfusion at day 7 (n=25) (Chemo+Tx). Blood was collected at day 21 and serum was evaluated for donor specific antibodies by flow cytometry **(B)** BALB/cJ splenic T cells were used as target cells, and bound antibody was detected with anti-Igκ (total allo-antibody) or with antibodies against specific isotypes (IgM, IgG1, IgG2b, IgG2c, and IgG3). The median fluorescent intensity for each mouse is plotted by isotype, with error bars showing mean and standard deviation. Data from 4 experiments were combined, samples from all experiments were screened at the same time on the same batch of splenocytes. One-way ANOVA with Tukey’s post-test was used to compare groups. *p<0.05, **p<0.01, ***p<0.001, ****p<0.0001.

To evaluate the impact of chemotherapy on the alloresponse to platelet transfusion, we utilized an existing model of AML chemotherapy developed by Zuber et al. (32) in combination with our own model of platelet transfusion alloimmunization (33–37). C57Bl/6J mice were given cytarabine with doxorubicin for 3 days (days 0-2) followed by 2 additional days of cytarabine alone (days 3-4), followed by transfusion of BALB/cJ non-leukoreduced platelets on day 7 (**Figure 2A**). Control groups included untreated mice, those given the chemotherapy protocol alone, or those given the transfusion alone. Circulating anti-donor antibodies were evaluated at day 21 (2 weeks after transfusion) by flow cytometry looking at total allo-antibodies (Igκ) or by isotype for IgM, IgG1, IgG2b, IgG2c, and IgG3. Surprisingly, we found that total anti-donor antibody levels were significantly higher in mice that were transfused following chemotherapy compared with transfusion in the absence of chemotherapy (**Figure 2B**). These differences were seen across isotypes with significantly higher levels of IgM and IgG1, and trends towards higher levels for IgG2b, IgG2c, and IgG3 in the chemotherapy and transfusion group compared to transfusion alone.

**Figure 3:**
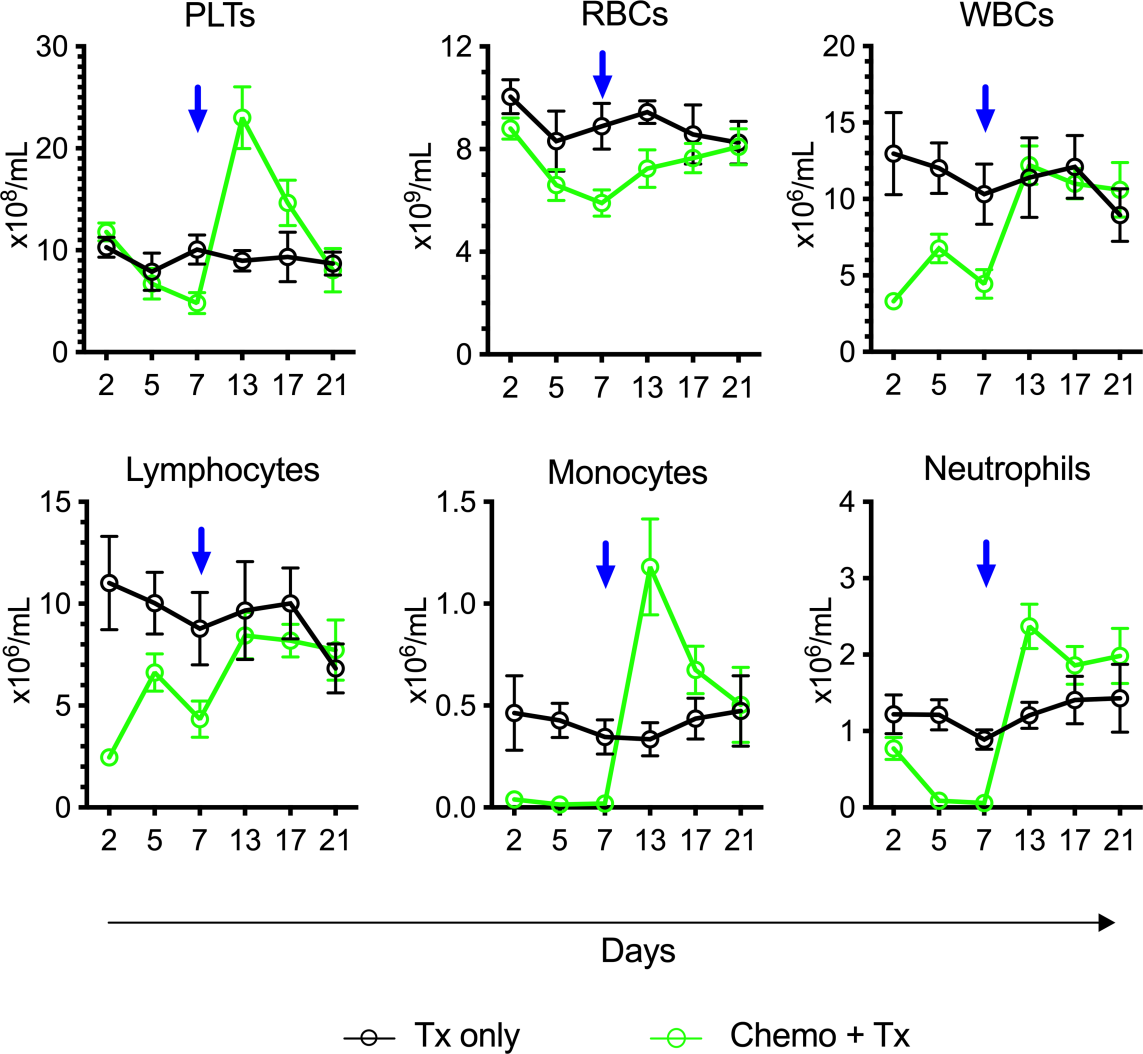
Lymphocytes in expansion phase at time of platelet transfusion. C57Bl/6J mice were given a 5-day course of cytarabine/doxorubicin days 0-4 (green) or no treatment (black), then transfused with BALB/cJ platelet at day 7 (blue arrow). Blood was sampled at days 2, 5, 7, 13, 17, and 21, and complete blood counts were run. Counts are given for platelets (PLTs), red blood cells (RBCs), white blood cells (WBCs), lymphocytes, monocytes, and neutrophils. Mean and 95% CI are plotted, n=10 per group.

To assess the degree and kinetics of ablation following this chemotherapy protocol, CBCs were evaluated over time from mice receiving chemotherapy and transfusion compared with transfusion alone (**Figure 3**). The timing of transfusion at day 7 corresponded to platelet and red blood cell nadir, but lymphocytes were depleted much earlier and were already in an expansion phase at the time of allogeneic exposure.

**Figure 4:**
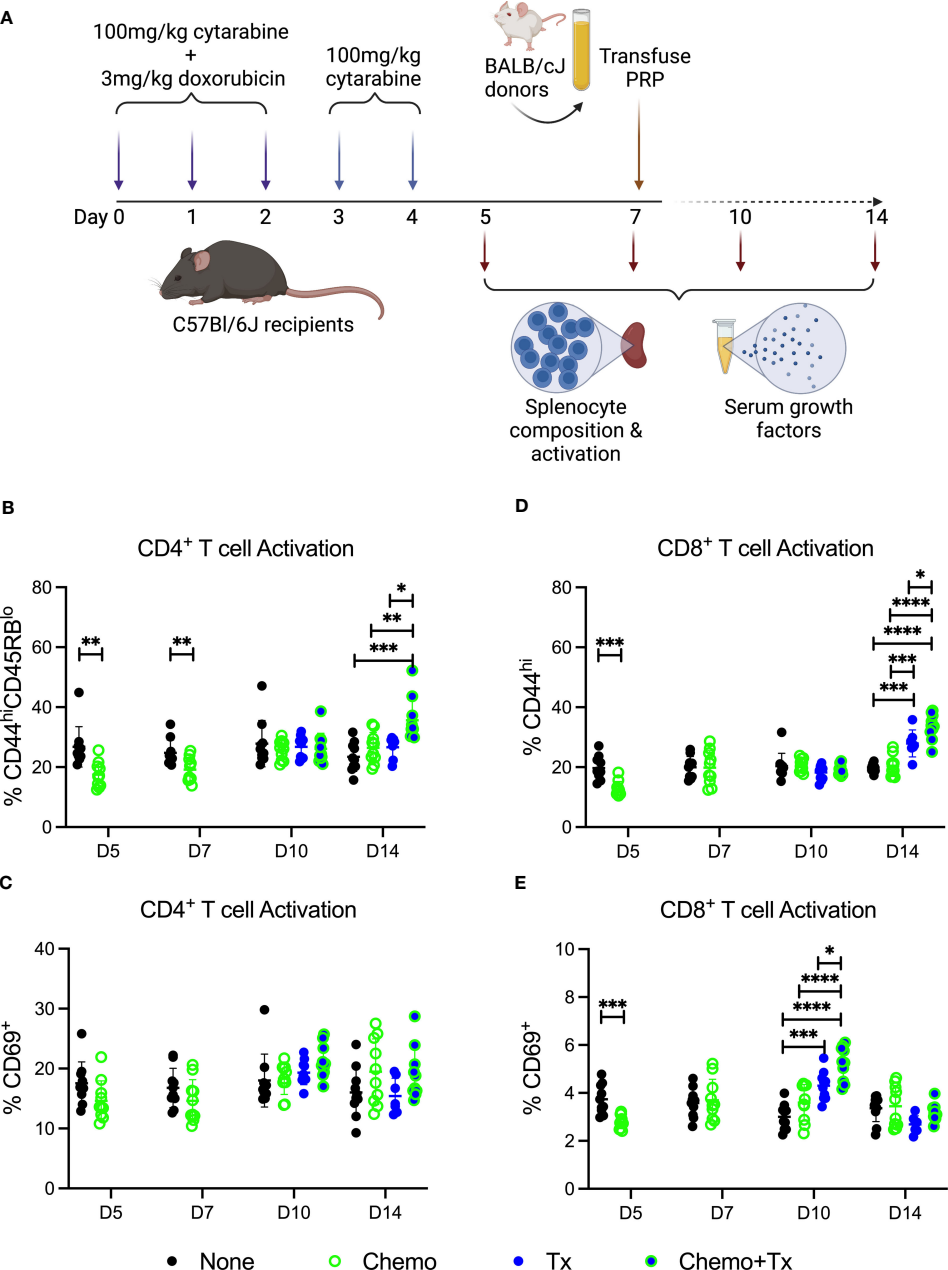
Chemotherapy increases T cell response to allogeneic platelets. **(A)** C57Bl/6J mice were given a 5-day course of cytarabine/doxorubicin days 0-4 (Chemo, green), no treatment (None, black), a BALB/cJ platelet transfusion at day 7 (Tx, blue), or the chemotherapy on days 0-4 followed by transfusion at day 7 (Chemo+Tx, blue/green). Spleens were harvested on days 5, 7, 10, or 14, and cells were evaluated by flow cytometry. Blood was also collected at these same time points with serum frozen for later analysis of growth factors. Cells were gated on singlets, lymphocytes, live cells, CD3^+^CD19^-^ cells, and then either CD4^+^ or CD8^+^ (see **Figures 1A–C** for gating). Frequencies of activated T cell populations are plotted. **(B)** Frequency of CD44^hi^CD45RB^low^ cells within the CD4^+^ T cell population. **(C)** Frequency of CD69^+^ cells within the CD4^+^ T cell population. **(D)** Frequency of CD44^hi^ cells within the CD8^+^ T cell population. **(E)** Frequency of CD69^+^ cells within the CD8^+^ T cell population. Data is pooled from 2 independent experiments, n=10 per group per time point (except Tx only at day 14 where n=7). Groups were compared within each time point, with unpaired t-tests used for pairwise comparisons at days 5 and 7, and one-way ANOVA with Tukey’s post-test used for days 10 and 14. Error bars indicate mean and standard deviation. *p<0.05, **p<0.01, ***p<0.001, ****p<0.0001.

As reestablishment of homeostasis following ablation can induce a more permissive immunological environment, we next evaluated the impact of this therapy on the immune environment around the time of allogeneic exposure. C57Bl/6J mice were given the same protocol of chemotherapy and transfusion, but sacrificed at day 5, 7 (prior to transfusion), 10, or 14 (after transfusion) with splenocytes and serum harvested for evaluation of cellular responses and soluble growth factors, respectively (**Figure 4A**). Activation of CD4^+^ T cells was assessed by expression of CD44/CD45RB (**Figure 4B**) and CD69 (**Figure 4C**), and activation of CD8^+^ T cells was assessed by expression of CD44 (**Figure 4D**) and CD69 (**Figure 4E**). Prior to transfusion, slight decreases were seen in the background frequency of activated T cells (both CD4 and CD8) in the chemotherapy group, perhaps due to a greater susceptibility of already activated lymphocytes to the treatment. Following transfusion, allogeneic T cell responses were significantly increased in mice given chemotherapy first compared to transfusion alone, with significantly higher frequencies of CD44^hi^/CD45RB^lo^ CD4^+^ T cells and CD44^hi^ CD8^+^ T cells at day 14 (7 days post-transfusion) and of CD69^+^ CD8^+^ T cells at day 10 (3 days post-transfusion).

**Figure 5:**
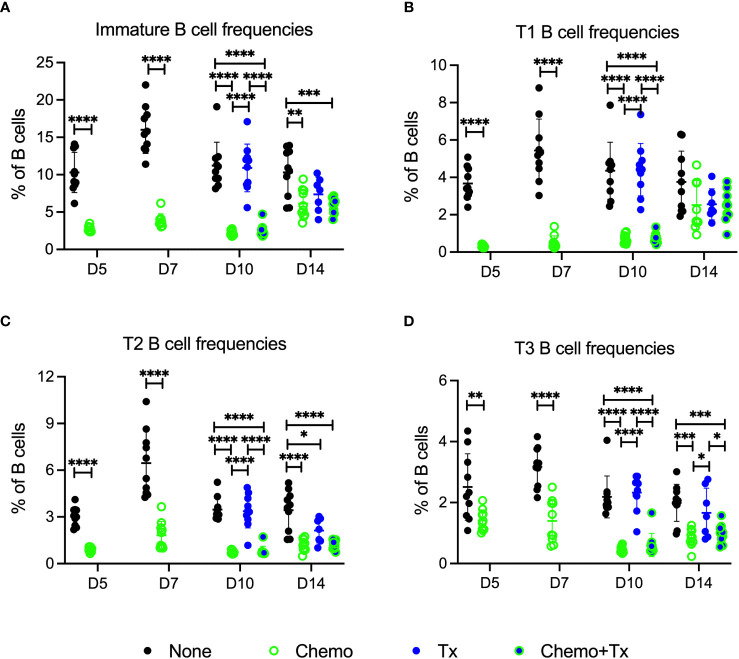
Chemotherapy disproportionately depletes less mature B cells. C57Bl/6J mice were given a 5-day course of cytarabine/doxorubicin days 0-4 (Chemo, green), no treatment (None, black), a BALB/cJ platelet transfusion at day 7 (Tx, blue), or the chemotherapy on days 0-4 followed by transfusion at day 7 (Chemo+Tx, blue/green). Spleens were harvested on days 5, 7, 10, or 14, and cells were evaluated by flow cytometry (See **Figure 4A**). Cells were gated on singlets, lymphocytes, live cells, and CD3^-^CD19^+^ cells to identify B cells. B cells were then further divided into mature (CD93^-^) and immature populations (CD93^+^), and immature B cells were further divided into T1 (CD23^-^/IgM^hi^), T2 (CD23^+^/IgM^hi^), and T3 (CD23^+^/IgM^low^) subsets (see **Figure 1A** for gating). **(A)** Frequency of immature B cell population out of the total B cell population. **(B)** Frequency of T1 B cell population out of the total B cell population. **(C)** Frequency of T2 B cell population out of the total B cell population. **(D)** Frequency of T3 B cell population out of the total B cell population. Data is pooled from 2 independent experiments, n=10 per group per time point (except Tx only at day 14 where n=7). Values are plotted along with mean and SD by group and time point. Groups were compared within each time point, with unpaired t-tests used for pairwise comparisons at days 5 and 7, and one-way ANOVA with Tukey’s post-test used for days 10 and 14. Error bars indicate mean and standard deviation. *p<0.05, **p<0.01, ***p<0.001, ****p<0.0001.

**Figure 6:**
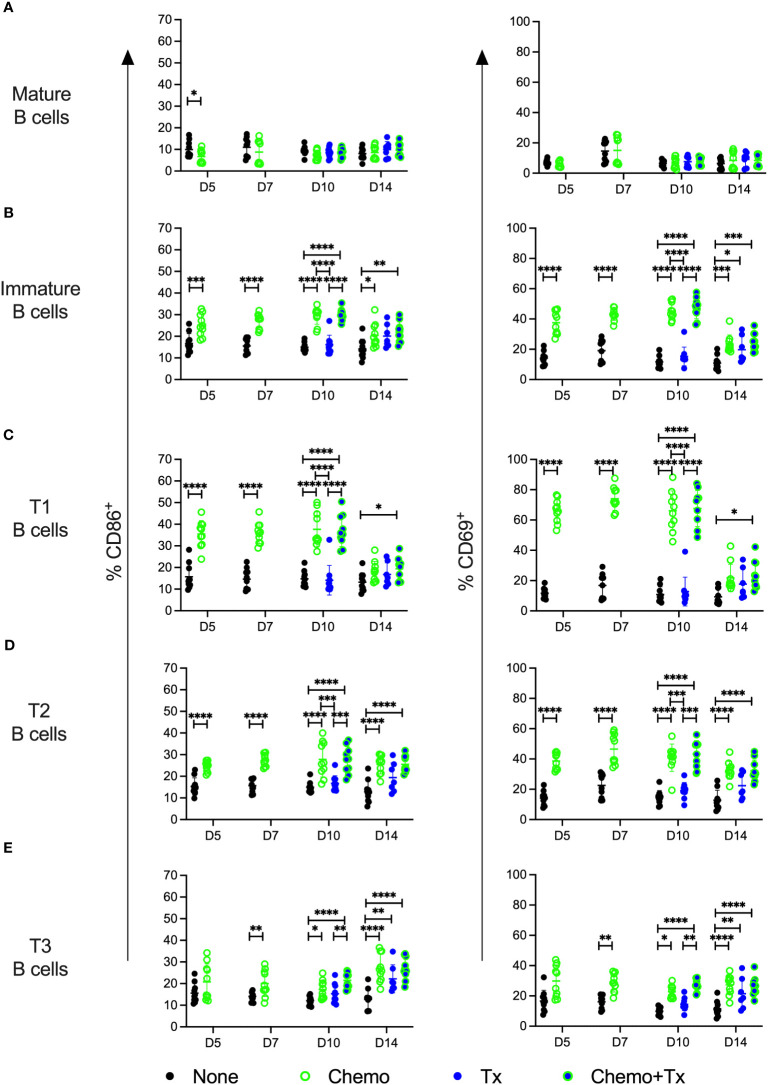
Chemotherapy induces non-specific activation of immature B cells. C57Bl/6J mice were given a 5-day course of cytarabine/doxorubicin days 0-4 (Chemo, green), no treatment (None, black), a BALB/cJ platelet transfusion at day 7 (Tx, blue), or the chemotherapy on days 0-4 followed by transfusion at day 7 (Chemo+Tx, blue/green). Spleens were harvested on days 5, 7, 10, or 14, and cells were evaluated by flow cytometry (See **Figure 4A**). Cells were gated on singlets, lymphocytes, live cells, and CD3^-^/CD19^+^ cells to identify B cells. B cells were then further divided into mature (CD93^-^) and immature populations (CD93^+^), and immature B cells were further divided into T1 (CD23^-^/IgM^high^), T2 (CD23^+^/IgM^high^), and T3 (CD23^+^/IgM^low^) subsets. Activation was determined based on upregulation of CD86 and CD69 expression. Total frequencies of CD86^+^ (left) and CD69^+^ (right) cells within gated Mature B **(A)**, total Immature B **(B)**, T1 B **(C)**, T2 B **(D)**, and T3 B **(E)** cells are shown (see **Figure 1A**, D for gating). Data is pooled from 2 independent experiments, n=10 per group per time point (except Tx only at day 14 where n=7). Values are plotted along with mean and SD by group and time point. Groups were compared within each time point, with unpaired t-tests used for pairwise comparisons at days 5 and 7, and one-way ANOVA with Tukey’s post-test used for days 10 and 14. Error bars indicate mean and standard deviation. *p<0.05, **p<0.01, ***p<0.001, ****p<0.0001.

The relative frequencies of mature (CD93^-^/CD24^lo^) versus immature/transitional (CD93^+^/CD24^hi^) peripheral B cells was measured, and a disproportionate depletion of the immature B cells was observed, with partial recovery by day 14 (**Figure 5A**). This depletion was observed across all 3 transitional B cell subsets but was most pronounced early on within the least mature T1 subset; T1 depletion resolved by day 14, while more mature T2 and T3 subsets resolved later, consistent with early depletion of precursors in the bone marrow (**Figures 5B–D**). Activation of each B cell subset was evaluated by expression of CD86 and CD69. For mature B cells, no significant differences in activation were observed with the exception of a slight decrease in CD86^+^ B cells at day 5 for the chemotherapy group, similar to what was observed for T cells (**Figure 6A**). In addition, no differences in activation were seen with the marginal zone or follicular B cell subsets either (data not shown). In contrast, the immature B cell population showed a high degree of non-specific activation following chemotherapy across all time-points, with no further enhancement of this response with the addition of transfusion, and only a weak response to transfusion alone (**Figure 6B**). This non-specific activation was most pronounced within the T1 population (**Figure 6C**) but was also observed within the T2 and T3 populations (**Figures 6D, E**).

**Figure 7:**
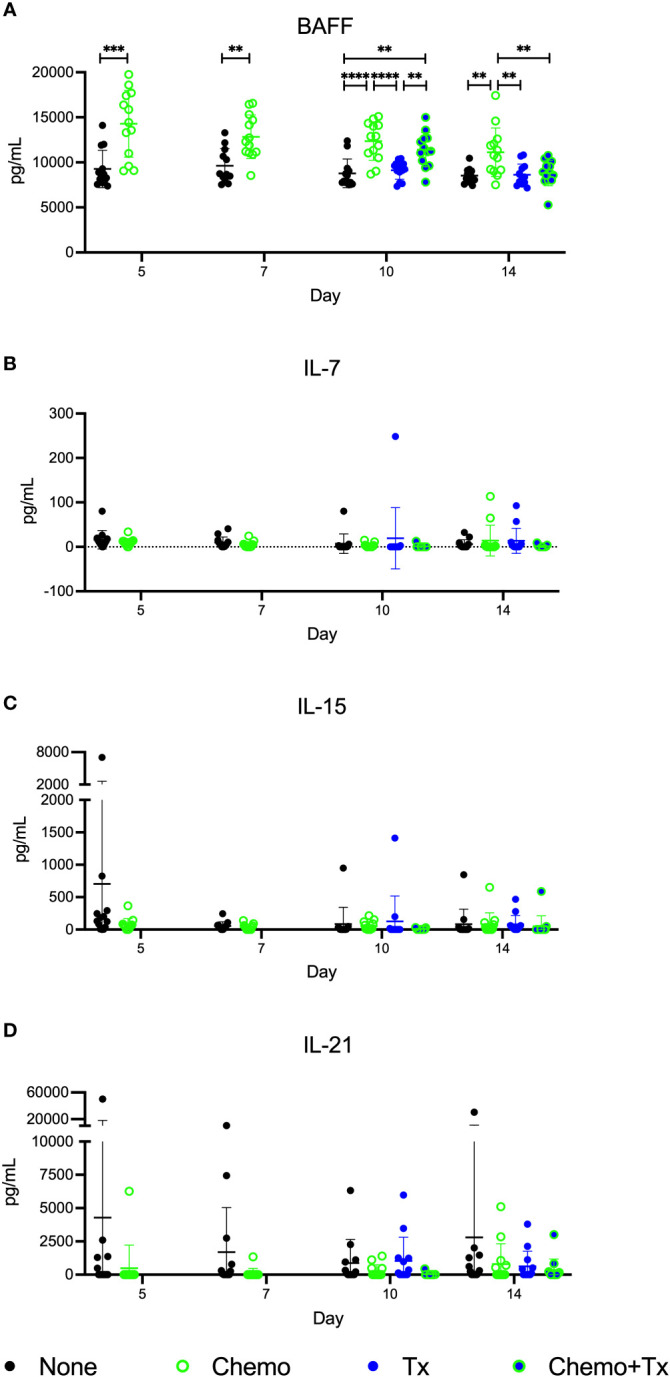
Circulating BAFF elevated following chemotherapy. C57Bl/6J mice were given a 5-day course of cytarabine/doxorubicin days 0-4 (Chemo, green), no treatment (None, black), a BALB/cJ platelet transfusion at day 7 (Tx, blue), or the chemotherapy on days 0-4 followed by transfusion at day 7 (Chemo+Tx, blue/green). Blood was collected on days 5, 7, 10, or 14 (See **Figure 4A**). Serum was screened for **(A)** BAFF by ELISA and for **(B)** IL-7, **(C)** IL-15, and **(D)** IL-21 by multiplex assay. Data is pooled from 3 independent experiments, n=13 per group per time point. Values are plotted along with mean and SD by group and time point. Groups were compared within each time point, with unpaired t-tests used for pairwise comparisons at days 5 and 7, and one-way ANOVA with Tukey’s post-test used for days 10 and 14. **p<0.01, ***p<0.001, ****p<0.0001.

Levels of the homeostatic growth factors IL-7, IL-15, IL-21, and BAFF were measured in the serum. No differences were found in IL-7, IL-15, or IL-21, but levels of BAFF were significantly elevated following chemotherapy with the greatest differences seen at day 5, and levels gradually reducing over time (**Figure 7**). Levels were still elevated at day 14 in the chemotherapy only group, but the chemotherapy and transfusion group was back to baseline, suggesting enhanced consumption of excess BAFF following alloantigen exposure.

**Figure 8:**
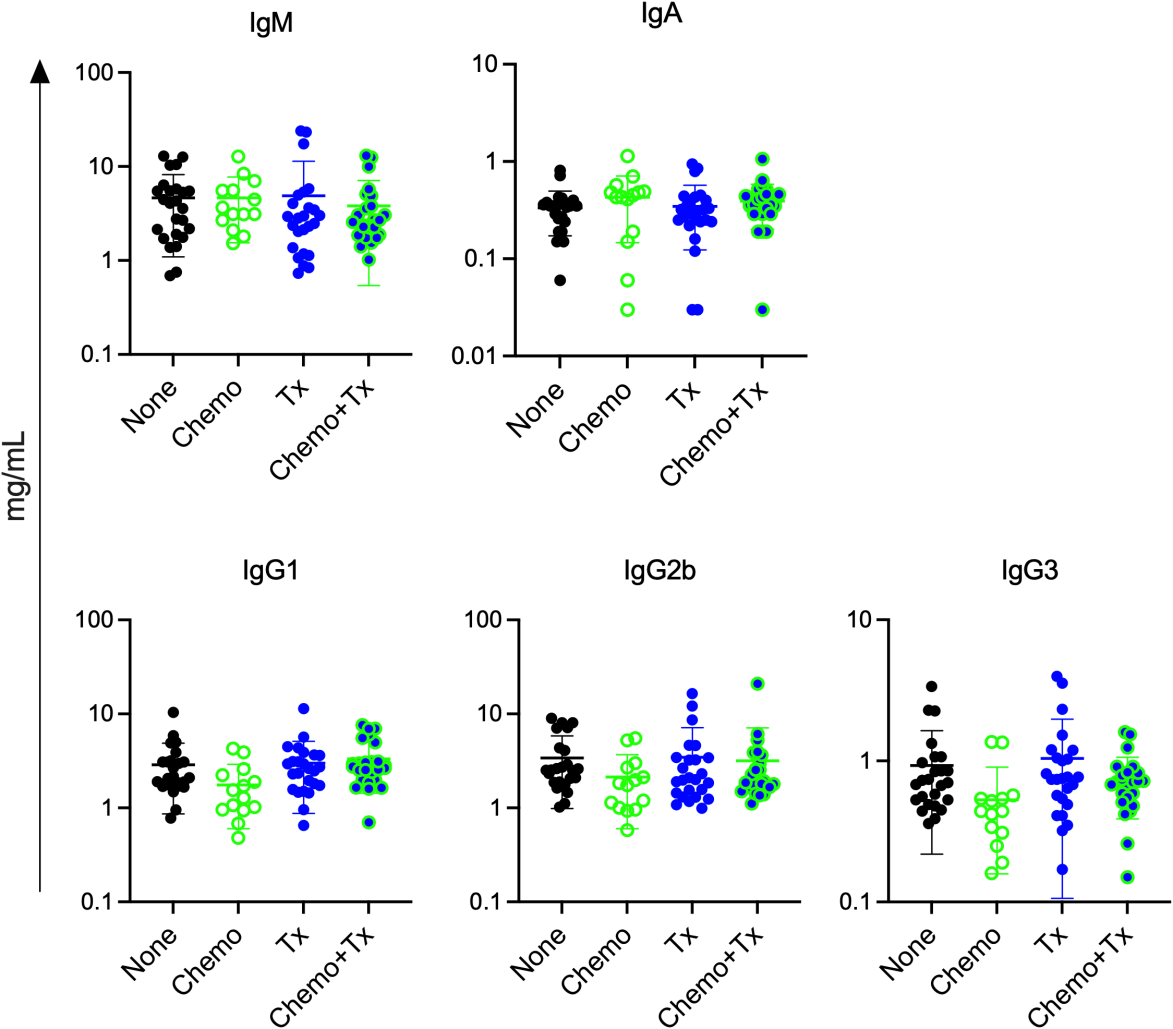
Chemotherapy does not increase total antibody levels. C57Bl/6J mice were given a 5-day course of cytarabine/doxorubicin days 0-4 (Chemo, n=14), no treatment (None, n=25), a BALB/cJ platelet transfusion at day 7 (Tx, n=25), or the chemotherapy on days 0-4 followed by transfusion at day 7 (Chemo+Tx, n=25). Blood was collected at day 21 (see **Figure 2A**). Serum was screened for total antibody by isotype using a multiplex assay. Concentration of total antibody for each mouse is plotted by isotype, with error bars showing mean and standard deviation. Data from 4 experiments were combined. One-way ANOVA with Tukey’s post-test was used to compare groups. No significant differences observed.

Since chemotherapy induced increases in activation of immature B cells regardless of alloantigen exposure, we next assessed if chemotherapy would lead to non-specific increases in circulating antibody levels. Levels of total IgM, IgA, IgG1, IgG2b, and IgG3 were measured in the same serum samples used to measure alloantibodies in **Figure 2B**. No significant differences were observed for any of the isotypes (**Figure 8**). This observation suggests that the non-specific activation of immature B cells did not result in overall increases in antibody levels, and that alloantibody production was specific to antigenic exposure.

## 4 Discussion

Contrary to our hypothesis, chemotherapy led to enhanced alloantibody responses to allogeneic platelet transfusion. These enhanced antibody responses were alloantigen specific and associated with increased T cell responses to alloantigen. Chemotherapy alone led to rapid depletion of lymphocytes, with heavy depletion of transitional B cells, followed by expansion and reestablishment of homeostasis. During this time, circulating levels of BAFF were elevated and transitional B cells were found to be non-specifically activated, with the highest degree of activation observed among the least mature T1 subset.

To our knowledge, the effect of chemotherapy on platelet alloimmunization has not been previously examined. The PLADO trial reported higher platelet alloimmunization in their chemotherapy only group compared with the stem cell transplant groups, but as the transplant groups would have also received ablative therapies, this is more of an association with transplant than chemotherapy (38). We do know that AML patients receiving chemotherapy can mount a robust alloimmune response. The TRAP trial focused entirely on this patient cohort and detected alloantibody generation in 13% of the recipients of unmodified platelet concentrates (7). Furthermore, attempts have been made to evaluate the impact of chemotherapy on RBC alloimmunization clinically with somewhat inconclusive results. Most of these studies have not seen significant differences associated with chemotherapy, but have struggled with confounding variables such as reduced disease severity and higher allogenic exposure within the no chemotherapy groups (39–41). One study, using the presence of a platelet transfusion as a proxy indicator of more severe chemotherapy, found reduced rates of alloimmunization to RBC antigens among the myeloproliferative and lymphoproliferative disease patients who were given platelets compared to those who were not (42). It is, however, difficult to know how much of this difference is attributable to chemotherapy as it was not looked at directly, and as with the other studies, is also associated with additional confounding variables. It is worth noting that chemotherapeutic approaches vary widely in effect, and other types of regimens (particularly non-ablative strategies) might alter alloimmunization differently.

The increased alloantibody responses observed following chemotherapy were not skewed towards a particular isotype, but rather, were seen across isotypes, suggesting that chemotherapy was not impacting class switch, but boosting antibody production overall (**Figure 2B**). While non-specific activation of transitional B cells was observed with chemotherapy alone (**Figure 6**), this was not associated with non-specific antibody production, as chemotherapy alone did not induce alloantibody production (**Figure 2B**) nor any increases in total antibody levels (**Figure 8**). This suggests that while non-specific activation of transitional B cells may occur with chemotherapy, antigen-specific signals are still required to induce an antibody response, even in this more permissive immunological environment. Interestingly, we did not observe any increased activation of mature B cells in any of our treatment groups, even when elevated antibodies were seen. Taken together, these data suggest that antibody production is coming from the transitional B cell populations. We cannot, however, rule out a role for mature B cells in this response, as we have observed in ongoing studies that the alloreactive B cell populations are rare, and that even in a robust alloimmune response, B cell activation is difficult to observe in the total B cell population, only becoming apparent with direct staining of alloreactive B cells. Additional studies will be needed to definitively determine the source of these alloantibody producing B cells.

The activation of the transitional B cells observed with chemotherapy is surprising, particularly as the T1 cells were the most activated subpopulation. Overall, transitional B cells have been shown to be more difficult to activate, with little to no proliferative response to anti-IgM or PMA and Ionomycin stimulation, and a reduced response to LPS stimulation compared with mature B cells, though they can generate T-dependent antigen-specific responses (27, 29, 31). A study from Petro et al. found that BCR signaling in the T1 subset led to cell death whereas in the T2 subset, this led to activation that was more similar to what is seen in mature B cells (30). In our study, all three of the transitional B cell subsets were activated in the chemotherapy groups, with the highest levels of activation seen in the T1>T2>T3 subsets. The key to this difference may be in the elevated BAFF we observed following chemotherapy. Overexpression of BAFF has been shown to induce autoimmune class-switched antibody production in mice in a transmembrane and CAML interactor (TACI)-dependent mechanism (43, 44). TACI, which is one of three known receptors for BAFF, is not normally expressed on most transitional B cells, but is found on a large subset of transitional B cells in BAFF transgenic mice, including the T1 subset (44). These TACI expressing transitional B cells have been shown in this high BAFF setting to be activated, enriched for autoreactivity, and capable of producing class switched autoantibodies (44).

In contrast to the non-specific activation seen in the transitional B cell compartment, increased T cell activation required allogeneic transfusion (**Figure 4**). Consistent with our earlier studies, we were able to detect activation of CD8^+^ T cells with transfusion alone (37). This response, however, was significantly increased in the chemotherapy and transfusion groups compared to transfusion alone. Our CD4^+^ T cell response was only significantly elevated with chemotherapy and transfusion together, suggesting that chemotherapy may be enabling greater T cell help for B cell responses to transfused alloantigens. This T cell help may be what is regulating the differences between the B cell activation markers seen with chemotherapy alone and the antibody increases observed with transfusion in the context of chemotherapy.

The homeostatic mechanisms regulating lymphocytes can generate niches for more permissive expansion when numbers of cells are low. When there are fewer cells to consume them, circulating levels of homeostatic growth factors can rise (22, 23, 45, 46). In our model, we see this clearly with elevated serum BAFF levels following chemotherapy (**Figure 7**). The elevated levels of BAFF are seen across the 2-week period we measured, but slowly reduce over time, consistent with lymphocyte recovery and increased consumption during this time. Furthermore, BAFF levels return to normal faster in the chemotherapy and transfusion group, suggesting that the introduction of an antigen-specific response leads to greater consumption of growth factors than homeostatic recovery alone. In addition to providing survival signals, BAFF signaling has been shown to play a role in the maturation of immature B cells to T1 cells and from T1 to T2 cells (47–49). Furthermore, as discussed above, high BAFF settings have been shown to induce activation within these transitional B cell subsets, consistent with our findings (44).

While ablative chemotherapies are generally thought of as immunosuppressive (20, 21), more evidence is accumulating that the effects of these treatments on immunological function are more complex. In recent years there has been a greater recognition of the immune-enhancing properties of traditional chemotherapies, with efforts to combine these approaches with immunotherapies to improve host responses targeting cancer cells. A number of mechanisms are thought to play a role including increasing the immunogenicity of the cancer antigens via more inflammatory cell death pathways, mucosal barrier injury leading to increased toll-like receptor signals, elimination of regulatory cell populations, and overall lymphodepletion leading to more permissive activation (50–52). While the antigen of interest in our model is derived from allogeneic platelets rather than cancer cells, many of these mechanisms may still play a role in altering the alloresponse to transfusion.

While our study has demonstrated that enhanced alloimmunization to platelets can occur following ablative chemotherapy, some limitations should be considered. While the use of a mouse model has enabled a reductionist approach that would not be feasible clinically, these findings should be verified in a clinical setting. We use non-leukoreduced platelets in our model as these produce a more robust alloresponse which is easier to track and manipulate than the response to leukoreduced platelets. Based on our previous work, we would anticipate a similar relative change in alloantibody responses using leukoreduced platelets (34, 35), but it is possible that the effects of chemotherapy on platelet alloimmunization outcomes is different with leukoreduction. Leukoreduction practices vary considerably leading to products with wide variation in both the number and type of residual lymphocytes found in the produced product, which may further alter alloimmunization outcomes (53). Furthermore, levels of expression of class I HLA on platelets can vary significantly by donor, potentially altering the impact of leukoreduction on alloimmunization from donor to donor (54). It is also important to note that transfusion at later timepoints post-chemotherapy might lead to different outcomes as homeostatic mechanisms gradually equilibrate. Finally, additional studies will be needed to determine the contribution of other factors such as different cancers and other therapeutic interventions typically given with ablative chemotherapy.

Alloimmunization to donor MHC can cause significant problems for platelet recipients including refractoriness to subsequent transfusions and rejection of transplants. As platelet transfusions and bone marrow transplants are more frequently given to patients on ablative therapies, understanding the risk of alloimmunization for these patients is critical. Our study suggests that ablative chemotherapies may increase a recipient’s risk of alloimmunization. These patients may benefit from targeted interventions such as HLA matching that are currently utilized only after a patient becomes refractory, or in the future, from repurposing of approved immune regulatory therapeutics such as BAFF blockade to inhibit the formation of new alloantibodies. While further studies will be needed to determine if these findings can translate to the clinic, an improved understanding of which patients are at highest risk of alloimmunization will enable the best support of those requiring transfusion.

## Data availability statement

The raw data supporting the conclusions of this article will be made available by the authors, without undue reservation.

## Ethics statement

These animal studies were approved by Institutional Animal Care and Use Committee at Labcorp Early Development Laboratories Inc. These studies were conducted in accordance with local legislation and institutional requirements.

## Author contributions

RJ: Conceptualization, Formal Analysis, Funding acquisition, Investigation, Supervision, Writing – original draft. OD: Investigation, Writing – review & editing. BG: Investigation, Writing – review & editing. JT: Investigation, Supervision, Writing – review & editing. MT: Conceptualization, Methodology, Writing – review & editing. MM: Conceptualization, Supervision, Writing – review & editing.

## References

[1] Howard JE Perkins HA. The natural history of alloimmunization to platelets. Transfusion (1978) 18(4):496–503. doi: 10.1046/j.1537-2995.1978.18478251250.x 684804

[2] Itescu S Tung TC Burke EM Weinberg A Moazami N Artrip JH. Preformed IgG antibodies against major histocompatibility complex class II antigens are major risk factors for high-grade cellular rejection in recipients of heart transplantation. Circulation (1998) 98(8):786–93. doi: 10.1161/01.CIR.98.8.786 9727549

[3] Massad MG Cook DJ Schmitt SK Smedira NG McCarthy JF Vargo RL. Factors influencing HLA sensitization in implantable LVAD recipients. Ann Thorac Surg (1997) 64(4):1120–5. doi: 10.1016/S0003-4975(97)00807-2 9354538

[4] Moazami N Itescu S Williams MR Argenziano M Weinberg A Oz MC. Platelet transfusions are associated with the development of anti-major histocompatibility complex class I antibodies in patients with left ventricular assist support. J Heart Lung Transplant (1998) 17(9):876–80. 9773859

[5] Slichter SJ Davis K Enright H Braine H Gernsheimer T Kao KJ. Factors affecting posttransfusion platelet increments, platelet refractoriness, and platelet transfusion intervals in thrombocytopenic patients. Blood (2005) 105(10):4106–14. doi: 10.1182/blood-2003-08-2724 PMC1895076 15692069

[6] Tsau PH Arabia FA Toporoff B Paramesh V Sethi GK Copeland JG. Positive panel reactive antibody titers in patients bridged to transplantation with a mechanical assist device: risk factors and treatment. ASAIO J (1998) 44(5):M634–7. doi: 10.1097/00002480-199809000-00067 9804512

[7] Leukocyte reduction and ultraviolet B irradiation of platelets to prevent alloimmunization and refractoriness to platelet transfusions. The trial to reduce alloimmunization to platelets study group. N Engl J Med (1997) 337(26):1861–9. doi: 10.1056/NEJM199712253372601 9417523

[8] Andreu G Dewailly J Leberre C Quarre MC Bidet ML Tardivel R. Prevention of HLA immunization with leukocyte-poor packed red cells and platelet concentrates obtained by filtration. Blood (1988) 72(3):964–9. doi: 10.1182/blood.V72.3.964.bloodjournal723964 3416079

[9] Dutcher JP Schiffer CA Aisner J Wiernik PH. Alloimmunization following platelet transfusion: the absence of a dose-response relationship. Blood (1981) 57(3):395–8. doi: 10.1182/blood.V57.3.395.bloodjournal573395 7459428

[10] Fauchet R Genetet B Gueguen M Leguerrier A Rioux C Logeais Y. Transfusion therapy and HLA antibody response in patients undergoing open heart surgery. Transfusion (1982) 22(4):320–2. doi: 10.1046/j.1537-2995.1982.22482251219.x 6179268

[11] Fisher M Chapman JR Ting A Morris PJ. Alloimmunisation to HLA antigens following transfusion with leucocyte-poor and purified platelet suspensions. Vox sanguinis (1985) 49(5):331–5. doi: 10.1111/j.1423-0410.1985.tb00807.x 3909636

[12] Murphy MF Metcalfe P Thomas H Eve J Ord J Lister TA. Use of leucocyte-poor blood components and HLA-matched-platelet donors to prevent HLA alloimmunization. Br J Haematol (1986) 62(3):529–34. doi: 10.1111/j.1365-2141.1986.tb02965.x 3954967

[13] Schiffer CA Dutcher JP Aisner J Hogge D Wiernik PH Reilly JP. A randomized trial of leukocyte-depleted platelet transfusion to modify alloimmunization in patients with leukemia. Blood (1983) 62(4):815–20. doi: 10.1182/blood.V62.4.815.815 6349715

[14] van Marwijk Kooy M van Prooijen HC Moes M Bosma-Stants I Akkerman JW. Use of leukocyte-depleted platelet concentrates for the prevention of refractoriness and primary HLA alloimmunization: a prospective, randomized trial. Blood (1991) 77(1):201–5. doi: 10.1182/blood.V77.1.201.201 1984797

[15] Whitaker BI Rajbhandary S Harris A. The 2013 AABB blood collection, utilization, and patient blood management survey report. Bethesda, MD: AABB (2015). 10.1111/trf.13676 27301995

[16] Grossman SA Ellsworth S Campian J Wild AT Herman JM Laheru D. Survival in patients with severe lymphopenia following treatment with radiation and chemotherapy for newly diagnosed solid tumors. J Natl Compr Canc Netw (2015) 13(10):1225–31. doi: 10.6004/jnccn.2015.0151 PMC4778429 26483062

[17] Kuter DJ. Managing thrombocytopenia associated with cancer chemotherapy. Oncol (Williston Park) (2015) 29(4):282–94. 25952492

[18] Menetrier-Caux C Ray-Coquard I Blay JY Caux C. Lymphopenia in Cancer Patients and its Effects on Response to Immunotherapy: an opportunity for combination with Cytokines? J Immunother Cancer (2019) 7(1):85. doi: 10.1186/s40425-019-0549-5 30922400 PMC6437964

[19] Wu Y Aravind S Ranganathan G Martin A Nalysnyk L. Anemia and thrombocytopenia in patients undergoing chemotherapy for solid tumors: a descriptive study of a large outpatient oncology practice database, 2000-2007. Clin Ther (2009) 31 Pt 2:2416–32. doi: 10.1016/j.clinthera.2009.11.020 20110050

[20] Harris J Sengar D Stewart T Hyslop D. The effect of immunosuppressive chemotherapy on immune function in patients with Malignant disease. Cancer (1976) 37(2 Suppl):1058–69. doi: 10.1002/1097-0142(197602)37:2+<1058::AID-CNCR2820370813>3.0.CO;2-O 766953

[21] Nesher L Rolston KV. The current spectrum of infection in cancer patients with chemotherapy related neutropenia. Infection (2014) 42(1):5–13. doi: 10.1007/s15010-013-0525-9 23975584

[22] Williams KM Hakim FT Gress RE. T cell immune reconstitution following lymphodepletion. Semin Immunol (2007) 19(5):318–30. doi: 10.1016/j.smim.2007.10.004 PMC2180244 18023361

[23] Fry TJ Mackall CL. The many faces of IL-7: from lymphopoiesis to peripheral T cell maintenance. J Immunol (2005) 174(11):6571–6. doi: 10.4049/jimmunol.174.11.6571 15905493

[24] Mackall CL Punt JA Morgan P Farr AG Gress RE. Thymic function in young/old chimeras: substantial thymic T cell regenerative capacity despite irreversible age-associated thymic involution. Eur J Immunol (1998) 28(6):1886–93. doi: 10.1002/(SICI)1521-4141(199806)28:06<1886::AID-IMMU1886>3.0.CO;2-M 9645370

[25] Mackall CL Bare CV Granger LA Sharrow SO Titus JA Gress RE. Thymic-independent T cell regeneration occurs via antigen-driven expansion of peripheral T cells resulting in a repertoire that is limited in diversity and prone to skewing. J Immunol (1996) 156(12):4609–16. doi: 10.4049/jimmunol.156.12.4609 8648103

[26] Min B McHugh R Sempowski GD Mackall C Foucras G Paul WE. Neonates support lymphopenia-induced proliferation. Immunity (2003) 18(1):131–40. doi: 10.1016/S1074-7613(02)00508-3 12530982

[27] Allman DM Ferguson SE Cancro MP. Peripheral B cell maturation. I. Immature peripheral B cells in adults are heat-stable antigenhi and exhibit unique signaling characteristics. J Immunol (1992) 149(8):2533–40. 1383316

[28] Allman DM Ferguson SE Lentz VM Cancro MP. Peripheral B cell maturation. II. Heat-stable antigen(hi) splenic B cells are an immature developmental intermediate in the production of long-lived marrow-derived B cells. J Immunol (1993) 151(9):4431–44. 8409411

[29] Cancro MP Allman DM Hayes CE Lentz VM Fields RG Sah AP. B cell maturation and selection at the marrow-periphery interface. Immunologic Res (1998) 17(1-2):3–11. doi: 10.1007/BF02786425 9479562

[30] Petro JB Gerstein RM Lowe J Carter RS Shinners N Khan WN. Transitional type 1 and 2 B lymphocyte subsets are differentially responsive to antigen receptor signaling. J Biol Chem (2002) 277(50):48009–19. doi: 10.1074/jbc.M200305200 12356763

[31] Allman D Lindsley RC DeMuth W Rudd K Shinton SA Hardy RR. Resolution of three nonproliferative immature splenic B cell subsets reveals multiple selection points during peripheral B cell maturation. J Immunol (2001) 167(12):6834–40. doi: 10.4049/jimmunol.167.12.6834 11739500

[32] Zuber J Radtke I Pardee TS Zhao Z Rappaport AR Luo W. Mouse models of human AML accurately predict chemotherapy response. Genes Dev (2009) 23(7):877–89. doi: 10.1101/gad.1771409 PMC2666344 19339691

[33] Jackman RP Heitman JW Muench MO. A small allelic variant in donor class I MHC is sufficient to induce alloantibodies following transfusion of standard or pathogen-reduced platelets in mice. Vox sanguinis (2020) 115(5):367–76. doi: 10.1111/vox.12897 PMC7374020 32201962

[34] Jackman RP Muench MO Heitman JW Inglis HC Law JP Marschner S. Immune modulation and lack of alloimmunization following transfusion with pathogen-reduced platelets in mice. Transfusion (2013) 53(11):2697–709. doi: 10.1111/trf.12133 23451715

[35] Muench MO Heitman JW Inglis H Fomin ME Marschner S Goodrich RP. Reduced alloimmunization in mice following repeated transfusion with pathogen-reduced platelets. Transfusion (2016) 56(6):1419–29. doi: 10.1111/trf.13579 27028210

[36] Tran JQ Muench MO Heitman JW Jackman RP. Allogeneic major histocompatibility complex antigens are necessary and sufficient for partial tolerance induced by transfusion of pathogen reduced platelets in mice. Vox sanguinis (2019) 114(3):207–15. doi: 10.1111/vox.12756 PMC6465144 30734299

[37] Tran JQ Muench MO Jackman RP. Pathogen-reduced PRP blocks T-cell activation, induces Treg cells, and promotes TGF-beta expression by cDCs and monocytes in mice. Blood Adv (2020) 4(21):5547–61. doi: 10.1182/bloodadvances.2020002867 PMC7656940 33166410

[38] Hess JR Trachtenberg FL Assmann SF Triulzi DJ Kaufman RM Strauss RG. Clinical and laboratory correlates of platelet alloimmunization and refractoriness in the PLADO trial. Vox sanguinis (2016) 111(3):281–91. doi: 10.1111/vox.12411 27185561

[39] Blumberg N Peck K Ross K Avila E. Immune response to chronic red blood cell transfusion. Vox sanguinis (1983) 44(4):212–7. doi: 10.1111/j.1423-0410.1983.tb01886.x 6601881

[40] Schonewille H de Vries RR Brand A. Alloimmune response after additional red blood cell antigen challenge in immunized hematooncology patients. Transfusion (2009) 49(3):453–7. doi: 10.1111/j.1537-2995.2008.01980.x 19243541

[41] Solh Z Athale U Arnold DM Cook RJ Foley R Heddle NM. Transfusion-related alloimmunization in children: epidemiology and effects of chemotherapy. Vox sanguinis (2016) 111(3):299–307. doi: 10.1111/vox.12419 27231826

[42] Schonewille H Haak HL van Zijl AM. Alloimmunization after blood transfusion in patients with hematologic and oncologic diseases. Transfusion (1999) 39(7):763–71. doi: 10.1046/j.1537-2995.1999.39070763.x 10413286

[43] Figgett WA Deliyanti D Fairfax KA Quah PS Wilkinson-Berka JL Mackay F. Deleting the BAFF receptor TACI protects against systemic lupus erythematosus without extensive reduction of B cell numbers. J Autoimmun (2015) 61:9–16. doi: 10.1016/j.jaut.2015.04.007 26027434

[44] Jacobs HM Thouvenel CD Leach S Arkatkar T Metzler G Scharping NE. Cutting edge: BAFF promotes autoantibody production via TACI-dependent activation of transitional B cells. J Immunol (2016) 196(9):3525–31. doi: 10.4049/jimmunol.1600017 PMC4868625 27022196

[45] Carter LM Isenberg DA Ehrenstein MR. Elevated serum BAFF levels are associated with rising anti-double-stranded DNA antibody levels and disease flare following B cell depletion therapy in systemic lupus erythematosus. Arthritis Rheum (2013) 65(10):2672–9. doi: 10.1002/art.38074 23839909

[46] Cambridge G Isenberg DA Edwards JC Leandro MJ Migone TS Teodorescu M. B cell depletion therapy in systemic lupus erythematosus: relationships among serum B lymphocyte stimulator levels, autoantibody profile and clinical response. Ann Rheum Dis (2008) 67(7):1011–6. doi: 10.1136/ard.2007.079418 17962238

[47] Rowland SL Leahy KF Halverson R Torres RM Pelanda R. BAFF receptor signaling aids the differentiation of immature B cells into transitional B cells following tonic BCR signaling. J Immunol (2010) 185(8):4570–81. doi: 10.4049/jimmunol.1001708 PMC2950883 20861359

[48] Sasaki Y Casola S Kutok JL Rajewsky K Schmidt-Supprian M. TNF family member B cell-activating factor (BAFF) receptor-dependent and -independent roles for BAFF in B cell physiology. J Immunol (2004) 173(4):2245–52. doi: 10.4049/jimmunol.173.4.2245 15294936

[49] Schiemann B Gommerman JL Vora K Cachero TG Shulga-Morskaya S Dobles M. An essential role for BAFF in the normal development of B cells through a BCMA-independent pathway. Science (New York NY) (2001) 293(5537):2111–4. doi: 10.1126/science.1061964 11509691

[50] Cornen S Vivier E. Chemotherapy and tumor immunity. Science (New York NY) (2018) 362(6421):1355–6. doi: 10.1126/science.aav7871 30573614

[51] Galluzzi L Humeau J Buque A Zitvogel L Kroemer G. Immunostimulation with chemotherapy in the era of immune checkpoint inhibitors. Nat Rev Clin Oncol (2020) 17(12):725–41. doi: 10.1038/s41571-020-0413-z 32760014

[52] Zitvogel L Apetoh L Ghiringhelli F Kroemer G. Immunological aspects of cancer chemotherapy. Nat Rev Immunol (2008) 8(1):59–73. doi: 10.1038/nri2216 18097448

[53] Semple JW Speck ER Cosgrave D Lazarus AH Blanchette VS Freedman J. Extreme leukoreduction of major histocompatibility complex class II positive B cells enhances allogeneic platelet immunity. Blood (1999) 93(2):713–20. doi: 10.1182/blood.V93.2.713 9885234

[54] Saris A Tomson B Brand A Mulder A Claas FH Lorinser J. Platelets from donors with consistently low HLA-B8, -B12, or -B35 expression do not undergo antibody-mediated internalization. Blood (2018) 131(1):144–52. doi: 10.1182/blood-2017-07-799270 29092829

